# Reversible Self-Actuated Thermo-Responsive Pore Membrane

**DOI:** 10.1038/srep39402

**Published:** 2016-12-19

**Authors:** Younggeun Park, Maria Paz Gutierrez, Luke P. Lee

**Affiliations:** 1 Department of Bioengineering, University of California at Berkeley, California, 94720, USA; 2 Berkeley Sensor and Actuator Center, University of California at Berkeley, California, 94720, USA; 3 Department of Architecture, University of California at Berkeley, California, 94720, USA; 4 Biophysics Graduate Program, University of California at Berkeley, California, 94720, USA; 5 Department of Electrical Engineering and Computer Science, University of California at Berkeley, California, 94720, USA

## Abstract

Smart membranes, which can selectively control the transfer of light, air, humidity and temperature, are important to achieve indoor climate regulation. Even though reversible self-actuation of smart membranes is desirable in large-scale, reversible self-regulation remains challenging. Specifically, reversible 100% opening/closing of pore actuation showing accurate responsiveness, reproducibility and structural flexibility, including uniform structure assembly, is currently very difficult. Here, we report a reversible, thermo-responsive self-activated pore membrane that achieves opening and closing of pores. The reversible, self-actuated thermo-responsive pore membrane was fabricated with hybrid materials of poly (N-isopropylacrylamide), (PNIPAM) within polytetrafluoroethylene (PTFE) to form a multi-dimensional pore array. Using Multiphysics simulation of heat transfer and structural mechanics based on finite element analysis, we demonstrated that pore opening and closing dynamics can be self-activated at environmentally relevant temperatures. Temperature cycle characterizations of the pore structure revealed 100% opening ratio at *T* = 40 °C and 0% opening ratio at *T* = 20 °C. The flexibility of the membrane showed an accurate temperature-responsive function at a maximum bending angle of 45°. Addressing the importance of self-regulation, this reversible self-actuated thermo-responsive pore membrane will advance the development of future large-scale smart membranes needed for sustainable indoor climate control.

Significant efforts have been placed in the last decade to develop smart membranes with self-actuation capabilities^1^,^2^, since energy and water savings are also some of the key resource advantages provided by smart membranes which allow us to achieve highly efficient ventilation and heat transmission, etc^3^,^4^,^5^. Smart membrane must selectively control the transfer of light, air, humidity, and temperature. Moreover, such responses operate in timely cycles according to the changes of stimuli to obtain punctual actuation while maintaining structural stability and uniformity especially in large scale. Smart membranes with pore actuation in response to environmental stimuli can provide such functions. For the pore actuation in the smart membrane, smart membranes have been developed to respond to stimuli. In these efforts, thermo-responsive^6^,^7^,^8^, magnetic^9^ and chemical species (ex. pH^10^,^11^, ions^12^, and specific molecules^13^,^14^) oriented actuations have been reported including thermo-responsive hybrid membranes^8^,^15^. In addition, there is a growing interest in biomimetics as ways of precise actuation of smart membrane structure so that they are more adaptable to the environment^16^,^17^. In nano/micro scale, the artificial cell membranes based on the biomimetics are also reported by chemically/physically incorporating stimuli-responsive materials as functional gates into traditional porous membranes. However, a large-scale smart membrane with adaptive capabilities remains highly challenging^18^,^19^. Since the fabrication of the current smart membrane structure is mostly based on incorporation or co-synthesis method which leads to less-controllable process^20^, irregular size of pores, lack of pore orderness, unclear open/close ratio of pores, stable actuation overtime, and mechanical stability of smart membranes remain as obstacles. Although, several lithography based previous efforts^21^,^22^,^23^ have shown single-dimensional membranes consisting of single materials (hydrogel only), localized actuation of the pore itself has not yet been demonstrated.

In response to this challenge, we are turning to nature to understand how skins function with effective multifunctional regulation capabilities. In the sub-micro scale, there are many examples in nature of breathable structures (not cells) with self-actuation properties^24^,^25^,^26^. One such example is plants. Plants can self-regulate air, heat, and internal humidity via micro-respirational pores which collectively open and thus can serve as a model for breathable membranes. Under conditions of light and elevated temperatures, leaves in plants absorb CO_2_ from the atmosphere through these respirational pores^25^,^27^. The role of light and temperature on the aperture mechanics of the respirational pore in plants is critical. The respirational pore, mediated by two guard cells, allows the exchange of water vapor and gases, such as CO_2_ and O_2_, through the transpiration process (Fig. 1). Specifically, the distinct, asymmetric geometry of the two guard cells plays a critical role in transpiration. The asymmetric geometry of the two guard cells reveals variation of diameter and nuclear location across its length. The guard cells are also tightly surrounded by epidermal cells. Under the conditions of light illumination and elevated temperatures, the guard cells absorb water and become swollen due to their ion channel (ex. K^+^) activation/inactivation in the cell membrane. As a result, the two guard cells bend apart from each other creating a micro pore opening for transpiration and gas exchange. In the absence of light illumination and ambient temperature, the pore closes. Thus, the two guard cells asymmetrically fold and unfold as a function of changes in temperature and light, allowing plants to self-regulate air, heat, and internal humidity^25^.

To our knowledge, there have been no demonstrations of smart membranes simultaneously showing reversible pore opening/closing with 100% on/off ratio, pore array uniformity, and steady actuation cycle. Well-defined single pore structures (*e.g.*, decoupled multi-dimensional membrane layers of protection and actuation materials, and ordered pore arrays) that enable reversible opening and closing abilities are necessary to advance the development of smart membranes. Here, inspired by the mechanical actuation of the plant respirational pore in response to changes in light and temperature, we report a reversible, self-activated thermo-responsive pore structure with varying three-dimensional pore size and pore height (Fig. 1). The self-activated multi-dimensional pore structure was fabricated by polymerization of poly (N-isopropylacrylamide), (PNIPAM) in the polytetrafluoroethylene (PTFE) body frame to form a hybrid structure. Using multiphysics simulation of heat transfer and structural mechanics based on finite element method, we show pore opening/closing dynamics which can be self-activated at environmentally relevant temperatures. Temperature cycle characterization of the pore structure revealed 100% opening ratio at *T* = 40 °C and 0% opening ratio at *T* = 20 °C. The flexibility of the membrane is demonstrated with accurate temperature-responsive function at a maximum bending angle of 45°. By addressing the challenges in self-regulating smart membranes, this self-activated, multi-dimensional pore structure will provide new opportunities in soft-matter functional materials, with the potential for practical implementation in built environments.

## Results and Discussions

**Figure 1:**
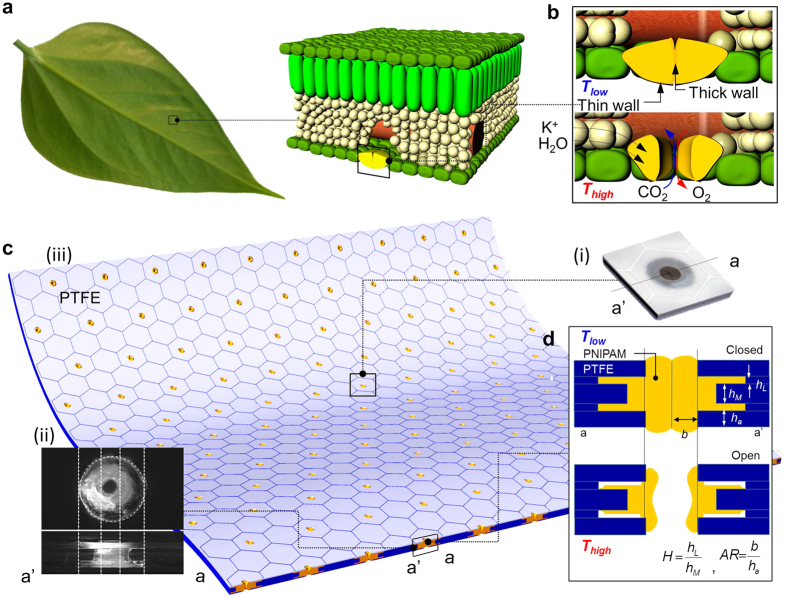
Inspirations from respirational pore structure of a plant leave to thermo-responsive pore opening and closing structure. (**a**) In nature, a plant leave which can regulate through respirational pore structure induced by light and temperature change; (**b**) The respirational pore opening and closing related to chemical and geometric asymmetric characteristics under light and temperature change; (**c**) Reversible self-actuated thermo-responsive pore membrane (i) Stereo microscopy image of single unit in the self-activated pore structure membrane, (ii) Top view and cross sectional view of pore structure taken by stereo microscopy, and (iii) Schematics of the reversible self-actuated thermo-responsive pore membrane with flexibility in large scale; (**d**) Temperature-dependent pore opening and closing schematics of the reversible self-actuated thermo-responsive pore membrane. By using principles (height difference in multilayered pore) found in plant respiration systems, the bioinspired self-activated pore structure exhibits self-activated actuation in response to temperature. The self-activated pore opens at high temperatures and closes at low temperatures. The uniform-sized pores are arrayed in an hexagonal pattern on the top layer of the self-activated pore structure. The variation in pore diameter in relation to structure thickness leads to multi-dimensional geometry, as with the respirational pore in plant leaves.

**Figure 2:**
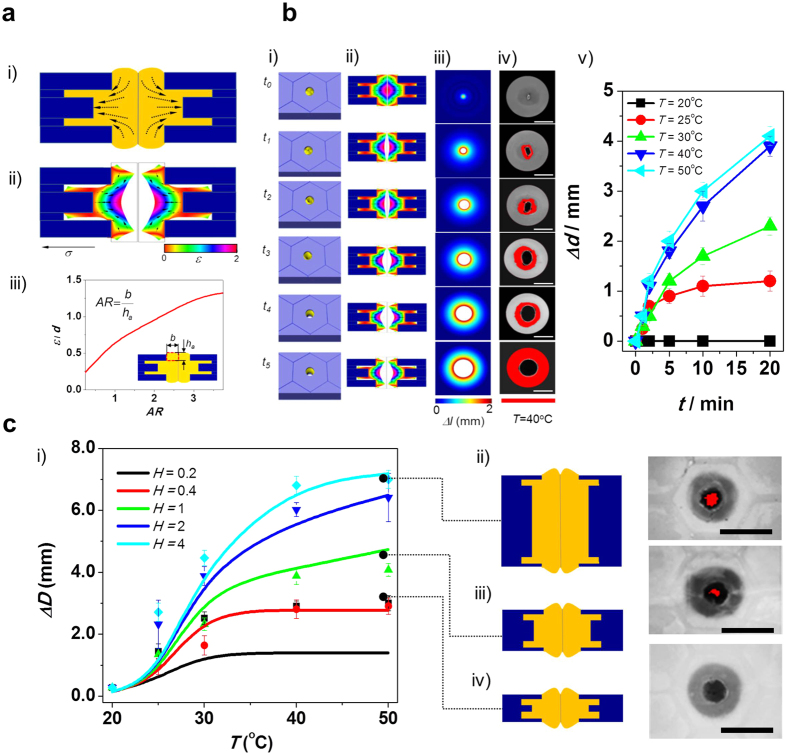
Systematic calculation and characterization of pore opening dynamics and the height difference effect on the displacement of the self-activated pore structure dependent on temperature. (**a**) Schematics of i) asymmetric geometry of self-activated pore, ii) stress and strain distribution in the self-activated pore structure, and iii) strain as afunction on top layer thickness. (**b**) i) schematics and image of the self-activated pore size as a function of time when the pore opens at higher temperature, ii) calculated dynamic stress and strain distribution, iii) calculated distribution of mechanical displacement from top view, iv) experimentally observed pore opening with heat distribution in the self-activated pore structure (scale bar = 5 mm) from top view. Red color indicates *T* = 40 °C in the structure and diffuses along the boundary of the opening pores with time after a change in temperature from *T* = 20 °C to *T* = 40 °C, and (v) Pore diameter change as a function of time in temperature variation at *H* = 4.0, AR = 2.0, *d*_*r1*_ = *0.5* and *d*_*r2*_ = *1.75* at *T* = 40 °C. (**c**) Geometry effect on pore opening; i) comparisons between calculated (solid line) and experimentally measured (dot) differential height (*H*) effect on the displacement of the structure at AR = 2.0, *d*_*r1*_ = *0.5* and *d*_*r2*_ = *1.75*, schematics and observed image at ii) *H* = 1.0, iii) *H* = 0.4, and vi) *H* = 0.2. Opened self-activated pore membrane image at *T* = 40 °C of vii) *H* = 4.0, viii) *H* = 2.0, and iv) *H* = 0.2. Red area stands for opened area (scale bar = 10 mm).

While the asymmetric shape of the respirational pore structure inspired us to design and fabricate reversible temperature-responsive self-activated pore structure, this study is not a mimicry of the respirational pore structure in plant leaves. Our first step in designing a self-activated pore structure was to computationally and experimentally verify our hypothesis that the geometrical differences between top/bottom and middle layers of a multidimensional structure will have a critical effect on the mechanical displacement in the pore structure as a result of temperature changes. First, we defined the geometry of the pore structure by varying its relative heights (*H* = *h*_*L*_*/h*_*M*_) and aspect ratio (AR = *b/h*_*a*_), including relative diameter 1 (*d*_*r1*_ = *d*_*E*_*/d*_*i*_) and 2 (*d*_*r2*_ = *d*_*o*_*/d*_*i*_) (Fig. 1 and Figure S1). As addressed in Fig. 1a, in a plant leaf, the physical actuation of the respirational pore is initiated by absorbing/desorbing water in the two guard cells in response to environmental energy changes. To demonstrate this phenomenon, we selected a PNIPAM hydrogel due to its temperature-responsive water absorption characteristics and hydration capability of its chemical structure^28^,^29^. PNIPAM is also a well-known biocompatible material and has a high potential for many practical applications. The driving force for the thermo-responsiveness of PNIPAM is entropy changes caused by absorption of water molecules from the hydrophobic alkyl-chain^30^,^31^,^32^. As temperature increases, dehydration of the CH_3_ groups, diffusion and aggregation of the chains, and the transition of hydrogen bonds takes place. Shrinkage is caused by the entropy elasticity of the PNIPAM network^33^,^34^. As a proof of concept, the pore images and temperature distributions for the self-activated pore structure as a function of time (Fig. 2 and Figure S2) revealed that the self-activated pore opens as a result of higher temperatures and thus, is temperature responsive. With the physical properties^35^,^36^,^37^, systematic finite element method (FEM) was conducted to estimate geometry change. A closed pore geometry at 20 °C was set as an initial condition for the self-activated pore structure. Then, a temperature change causes a mechanical displacement of the structure. In this analysis, as *T* increased from 20 °C to 50 °C, *Δd* (change in pore diameter) increased with temperature (Detailed simulation conditions are listed Table S1 and S2). In Fig. 2a, due to the asymmetric shape of the pore structure, shrinkage direction of the self-activated pore was focused at the center of the structure. Furthermore, this asymmetric shrinkage induced an interlocked structure without any dislocation from a body frame under higher temperature. In addition, theoretical results were compared to experimental results for pore opening dynamics (Fig. 2b, the red color indicates the zones of high temperature, based on the transparency of the PNIPAM). In this analysis, we visualized optical density changes of the structure to confirm the pore diameter changes at high temperature. At high temperatures, PNIPAM structure loses water from the chemical framework, causing a decrease in transparency of the PNIPAM structure. From this fact, the temperature-based water diffusion in the PNIPAM structure can be estimated from the transparency of the PNIPAM structure. We visualized the process of pore size changes with a geometry (*d*_*r1*_ = *0.75, d*_*r2*_ = 1.75, AR = 2.0 and *H* = 1.0) at *T* = 20 °C. The initial pore structure was initially closed. When the temperature shifted from *T* = 20 °C to *T* = 50 °C, pore opening occurred from the center to the side of the pore at time *t*_*1*_. This image revealed that the direction of stress converged evenly around the pore center, where thickness differences were observed starting from time *t*_*2*_. The structure then contracted at time *t*_*3*_, allowing the pore to open. In the self-activated pore structure, since the pore center was initially thicker than the boundary layer, the direction of structural contraction was continuous toward the center of the pore. This actuation trend is similar to respirational pore structures which have a high-density pore center in a plant leave^24^,^25^,^26^,^38^. As the last stage of this pore actuation, the thickness of the pore structure at the center becomes thinner and the pore opens. Pore actuation dynamics is temperature-dependent, as shown in a plot of pore opening as a function of time with temperature variation from T = 20 °C to T = 50 °C (Fig. 2b–v)). After showing agreement between computational and experimental demonstration of the self-activated pore, we refined the structural design and proceeded with the experimental fabrication of the self-activated pore structure. Subsequently, we compared pore opening between experimentally investigated data (dotted line) and simulation result (solid line) of the relative height, *H* effect on the pore opening at different temperatures as shown in Fig. 2c. Increased volume changes resulted in a large pore opening of up to 6.5 mm (at *H* = 4.0), while the pore opening in *H* = ~2.0 becomes less sensitive to the temperature changes. Pore openings between simulation and experimental result are almost matched in high *H*. Pore opening in the low *H* show less matching between simulation and experiment. Since water diffusion was not accounted for in the simulation, underestimation of the pore size change was observed at low *H*. In particular, at *T* = 50 °C and *H* = 4, a maximum value of *Δd* (=8.0) was obtained, while at *T* = 20 °C, *Δd* was nearly constant with *H* varying from 0.2 to 4. Notably, *H* = 4 led to a larger displacement than the pore diameter (*d*_*i*_ = 5 mm), which could potentially lead to structural deformation. These results indicated that *H* is the most important parameter for maximal pore opening without structural deformation, since changes in structural volume due to height variation lead to high stress gradient and strain. To confirm the *H* effect on the pore opening, we also estimated the pore diameter effect using pore diameter change as a relevant parameter (Figure S3). The inside pore diameter (*d*_*r1*_) was varied from 0.05 to 0.75, and maximum displacement was consistent around 4.0 mm at *T* = 50 °C. In addition, varying relative outside pore diameter (*d*_*r2*_) from 1.0 to 4.0 also showed that *Δd* was saturated at ~4.0 mm regardless of pore diameter change. Consequently, the pore structure change based on height differences has a strong effect on pore opening.

**Figure 3:**
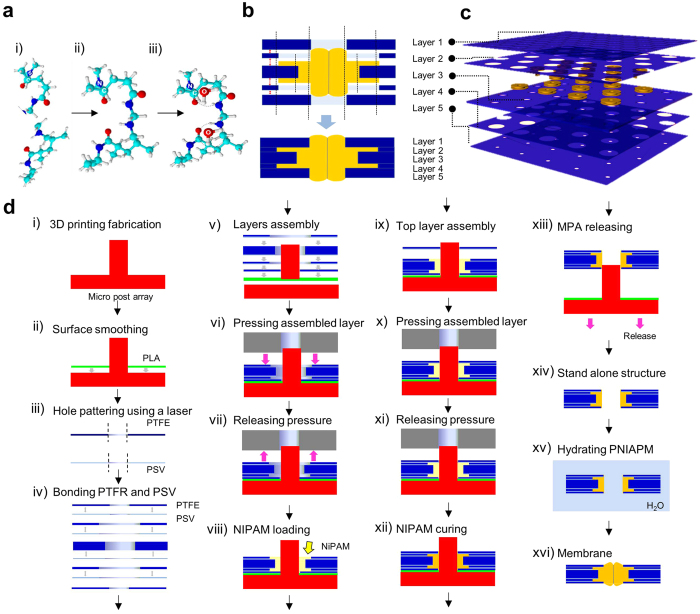
Structure and fabrication step for self-activated pore structure. (**a**) Chemical pathway of polymerization of NIPAM and hydration of the polymerized PNIPAM under water rich condition. (**b**) A body frame of the multidimensional PNIPAM pore structure ensured by assembly of 5 PTFE layers with different size of pore diameter. In the design, the PNIPAM pore structure is physically embedded in the PTFEs body frame. The PTFE body frame consists of 5 different layers; the smallest sized pores of the 1^st^ (Top) and 5^th^ (Bottom) PTFE layer seal the NIPAM pore. The largest sized pores of the 2^nd^ and 4^th^ PTFE layer hold the PNIPAM structure. In addition, the 3^rd^ PTFE layer consists of medium sized pores plays role of fixing the location the PNIPAM pore structure in the middle of the assembled PTFE layers. Due to the stacking of the PTFE multilayers with different pore size, the PNIPAM pore structure can be interlocked. The multidimensional PNIPAM in the PTFE structure can be actuated without any dislocation. The calculated strain distribution in the PNIPAM structure indicates the stable actuation by showing focused shrinkage around center. (**c**) Large area film assembly with multidimensional pore structure, and (**d**) Fabrication step: i) As a template for multilayer PTFE stacking, micro post array (MPA) is printed by a 3D printer; ii) top-surface of the template is attached by vinyl film to ensure hydrophilic characteristics; iii) Different sized pores in each PTFE layers are patterned by using a laser cutter. As a bonding layer, sensitive vinyl printing (PSV) sheets are also patterned by the laser cutter; iv) Before stacking, along alignment marks, the each PTFE is attached with each PSV layer consists of the same pore size; v) 4 PTFE layers (From layer 5 and layer 2) are stacked along the post array and the pre-patterned alignment mark on each PTFE layer, vi) The stacked PTFE layers are pressed by solid structure to ensure bonding strength between PTFE layers, vii) the solid structure is released; viii) NIPAM solution is loaded into hole formed by multilayer stacking; ix) Top layer (PTFE/PSV) is stacked on the pre-stacked PTFE layers (top PTFE film (layer-1) patterned with hexagonal array is attached on the assembled structure (layer 2–5) along the post array and the pre-printed alignment mark on the sheet); x) The stacked PTFE layers are pressed by solid structure to ensure bonding strength; xi) the solid structure is released; xii) NIPAM structure is thermally cured; xiii) MPA is removed from the assembled membrane structure; xiv) Standalone membrane body is inspected by stereo microscope to confirm design of the membrane; xv) At room temperature, the fabricated structure is incubated in high humidity condition to hydrate PNIPAM pore structure based on water absorption chemistry. The PNIPAM pores in the fabricated membrane are closed at room temperature; xvi) the membrane structure is activated to response environmental temperature change.

With the confirmation of the design parameter, we fabricated the self-activated pore structure membrane consisting of 5 PTFE layers with varying pore diameters (Fig. 3). Hydrophobic body frame was the key feature of this study. Due to the hydrophobicity of the body frame, when NIPAM solution was loaded into the hole of the PTFE, we can selectively load the hydrophilic NIPAM solution into the hole without spreading or leakage of NIPAM solution into gap between PTFE layers. Using our microfabrication approach, we did not observe issues with filling NIPAM monomer into the space of the PTFE pore. For the assembly of each layer, double sided pressure sensitive vinyl film (PSV) was used to ensure adhesion between PTFE and PNIPAM and easy assembly. After assembly and filling with NIPAM solution, thermal polymerization was conducted to create uniform PNIPAM pore structures in the PTFE body frame. As a result, the variation of pore diameter at each PTFE layer induced an asymmetric geometry of PNIPAM. In the case of many hybrid or multi-layered membranes, membrane disassembles can become an issue because of the different thermal expansion coefficients of the different materials in the membrane structure in response to temperature variation. However, since the asymmetric PNIPAM pore structure is placed within the multi-dimensional PTFE body frame as an interlocked structure, the PNIPAM pore structure is not disassembled even under temperature and mechanical stress. Specifically, the pore diameter of the 1^st^ and 5^th^ layer (top and bottom utmost layers) are smaller than the outside diameter of PNIPAM structure, so that the PNIPAM pore structure is physically embedded inside the PTFE pore. In addition, the pore diameter of the 3rd layer is also smaller than outside diameter of the PNIPAM structure, such that the cured PNIPAM was successfully placed within the PTFE pore without any chemical and thermal bonding. The 2nd and 3rd layer have the function of guiding the outside diameter of the PNIPAM. Through this multi-dimensional pore design, even with high volumetric change and shrinkage, PNIPAM pore structure successfully remains inside the PTFE body frame. This interlocked design allowed the structure to ensure its structural integrity regardless of swelling and shrinkage. In addition, the multi-dimensional PNIPAM structure induced an asymmetric shape change. Due to the thicker middle layer of PNIPAM, shrinkage direction was focused at the center of the structure as addressed in Fig. 2. This asymmetric shrinkage of the structure helped to maintain the structure in the PFTE body frame. To construct these 5 PTFE layers with 3 different pore diameters, at first, micro post array (MPA) was used as a template to guide the aligned assembly of the PTFE layers. Using a 3D printer, we printed the MPA structure. Each PTFE layers were fabricated by a laser cutter with different pore size. Before assembly of layers, bottom surface of the MPA array was covered by vinyl film to ensure interaction between MPA surface and PTFE layer. Then, 4 PTFE layers (from layer 2 to layer 5) with 3 different pore diameters were stacked along the aligned MPA by using pre-attached PSV film on each PTFE layer (Table S1–2). Depending on volume of the pore, a defined volume of NIPAM solution was loaded into the multidimensional pore space generated by PTFE layer assembly. Then, a top PTFE layer (layer 1) with pre-attached PSV layer was stacked to the top of assembled layers 2–5. As a final stage of fabrication, the assembled membrane was hydrated at room temperature to form a closed PNIPAM pore.

**Figure 4:**
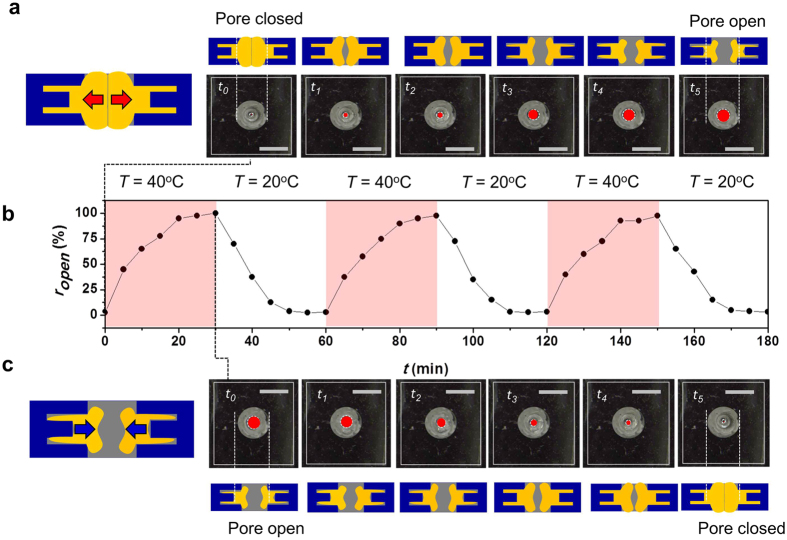
Cycle of pore opening and closing dependent on environmental temperature at *T* = 20 °C and *T* = 40 °C. (**a**) Time lapse photo images with schematics of pore opening in self-activated pore membrane structure (*d*_*r1*_ = *0.5, d*_*r2*_ = *1.75*, AR = 2.0 and *H* = 1.0) at T = 40 °C; (**b**) Pore opened ratio as function of time under temperature switching environment between *T* = 20 °C and *T* = 40 °C. Opened area pores, *r*_*open*_ (=opened area of pore/100% opened area of pore) with cycle of self-activated pore structure pore open and close at *d*_*r1*_ = *0.5, d*_*r2*_ = *1.75*, AR = 2.0 and *H* = 1.0 based on temperature cycle from *T* = 20 °C to *T* = 40 °C; (**c**) Time lapsed photo images of pore closing in the self-activated pore membrane structure (*d*_*r1*_ = *0.5, d*_*r2*_ = *1.75*, AR = 2.0 and *H* = 1.0) at *T* = 40 °C (Scale bar = 10 mm).

As the 1^st^ required for application of the self-activated pore structure, we characterized steady actuation capability with temperature change cycle. Thus, we monitored the pore open/close actuation cycle and stability in response to temperature change from 20 °C to 40 °C. In the pore actuation, three temperature-dependent steps were considered; 1) opening the pore, 2) maintaining the opened pore, and 3) closing the pore. Each step of the pore actuation cycle occurred at specific temperatures and time frames. For the actuation cycle test, the sample initially at *T* = 20 °C was moved to an environment at *T* = 40 °C and left for 30 min. Then, the environmental temperature was switched back to the cold condition (*T* = 20 °C). This cycle was repeated 5 times. In Fig. 4, the pore opening and closing (geometry: *d*_*r1*_ = 0.75, *d*_*r1*_ = 1.75, AR = 2.0 and *H* = 1.0) is shown between *T* = 20 °C and *T* = 40 °C. It took ~20 min to open 100% at *T* = 40 °C. In case of 100% closing, it took 10 min at *T* = 20 °C. Currently, no method yet has been reported to control pore opening/closing with 100% efficiency^21^,^22^,^29^,^39^. Furthermore, during actuation, no deformation of pore shape was observed over time. This result suggests that the self-activated pore is stable. The stability can be attributed to the residual strain of PNIPAM^28^,^37^,^40^ and the interlocked PNIPAM pore structure. In addition to verifying the changes in pore structure for the self-activated pore in response to temperature change, we also characterized the time in the cycles. As described in Fig. 2b, we analyzed the actuation response time within the temperature range of 20 °C to 50 °C. Interestingly, we observed that ~5 min lag exists until start of the actuation after the temperature change. This time lag may be due to water molecule diffusion in the PNIPAM structure. As temperature increased to *T* = 40 °C, the response time for pore opening was faster than for pore closing. Specifically, in the same 5 min time period, at *T* = 30 °C, the pore opened up to 0.75 mm, while at *T* = 40 °C, the pore opened to 1.5 mm. Generally, higher temperature gradient led to faster response time. In detail, faster response times can be explained by two driving forces in the mechanics of PNIPAM hydrogels: osmotic pressure and residual strain. Osmotic pressure of the porous PNIPAM is temperature-responsive, while residual strain is an innate property of the PNIPAM hydrogel itself. For temperatures higher than *T* = 40 °C, the alkyl chain of PNIPAM are no longer bonded with water molecules^30^,^31^,^32^. Thus, the osmotic pressure decreases and the residual strain becomes the predominant force governing the structural behavior mechanics of the hydrogel. Surprisingly, at high temperatures (>40 °C), the opened pore structure was maintained without further displacement. Preventing the further pore actuation (continuing to open) at high temperatures is important for the durability of the pore structure. Proper accumulative stresses in the pores structure is considered to be important to maintain the pore shape without further actuation for durability.

**Figure 5:**
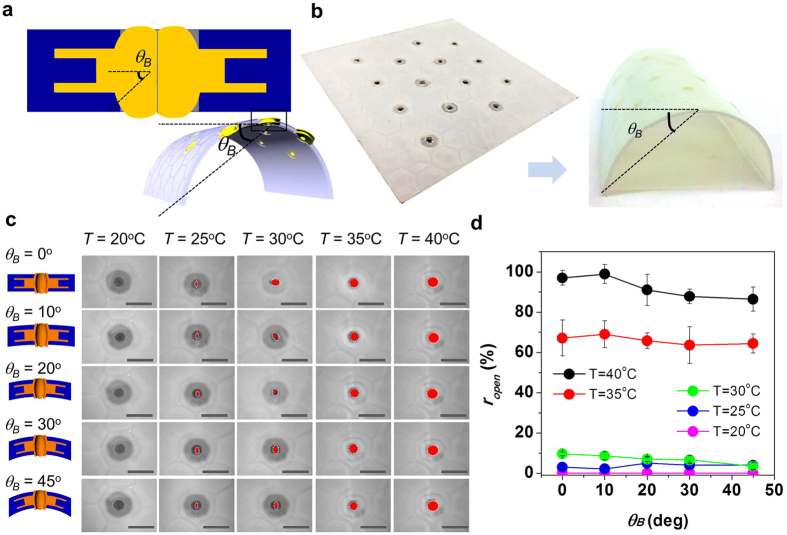
Stable pore actuation of the self-activated pore structure with mechanical bending under enviromental temperature changes from *T* = 20 °C to *T* = 40 °C. (**a**) Definition of bending angle (*θ*_*B*_) of the self-activated pore structure, (**b**) Comparison of photo images between flat and bent the self-activated thermo responsive pore structure, (**c**) Pore images as a function of bending from 0° to 45° at different temperature (*d*_*r1*_ = *0.5, d*_*r2*_ = *1.75*, AR = 2.0 and *H* = 1.0) (scale bar = 10 mm) with schematics of pore shape at each angle, (**d**) pore opened ratio (*r*_*open*_ = opened area of pore/100% opened area of pore) as a function of bending angle at different temperature at *d*_*r1*_ = *0.5, d*_*r2*_ = *1.75*, AR = 2.0 and *H* = 1.0.

With clear pore actuation dynamics and cycles in response to temperature change, we expected that consistent pore actuation for 100% opening/closing under large induced bending could be achievable, if the multi-dimensional PNIPAM pore structure is well-interlocked in the multilayered PTFE films. To confirm durability of the self-activated pore under structural bending, we tested the effect of structural bending on pore actuation under temperature change. The opening/closing of the pores in the self-activated pore structure was observed as a function of bending angle, *θ*_*B*_ from 0° to 45° between *T* = 20 °C and *T* = 40 °C with intervals of 5 °C (Fig. 5 and Figure S4). As expected, as *θ*_*B*_ increased, the pore actuation of opening/closing dynamics at *T* = 25 °C and *T* = 40 °C led to a 100% opened pore. Between *T* = 30 °C and *T* = 35 °C, ~70% of pore opening occurred. Even though the pore actuation was decreased in ~1% over *θ*_*B*_ = 30 °, the pore actuation under bending revealed consistent opening ratio. Again, the consistent and durable pore actuation with flexibility can be attributed to hybrid membrane structure of the interlocked PNIPAM pore structure in the PTFE multilayered body frame. For the future application, we will consider advanced smart hybrid membranes for atmospheric condition. In this regards, we anticipate that modified molecular structure of the hydrogel will be a next material step for future applications. Our next research phase will also consider the effects of humidity and temperature on structural changes with modified PNIPAM and different materials.

## Conclusions

In conclusion, we have designed and demonstrated a reversibly actuated, three-dimensional pore membrane structure that can respond to temperature changes. This thermo-responsive pore structure is capable of controlling potential ventilation rates by the opening and closing of the pores. Through systematic design and simulation approaches, we demonstrated the pore geometry is crucial to control the opening/closing dynamics of the reversible, self-actuated pore membrane in response to temperature shifts. We fabricated the optimized, reversible self-actuated thermo-response pore membrane structure based on a hybrid material design of PNIPAM within PTFE. The multiphysics-based heat transfer and structural mechanics simulation of the reversible self-actuated pore membrane structure revealed 100% opening of its pore at *T* = 40 °C. The fabricated structure showed 100% pore opening/closing through temperature change from *T* = 20 ^ο^C to *T* = 40 ^ο^C in approximately 20 min, and the self-and reversibly-actuated pore membrane showed distinctive cycles of pore opening/closing triggered by temperature changes. We demonstrated structural flexibility of the reversible, self-actuated pore membrane at a maximum bending angle of 45°. We envision that the reversible, thermo-responsive and self-activated pore membrane will contribute to future large-scale membranes with self-regulating capabilities for sustainable indoor climate control.

## Method

### Experimental section

#### Materials

N-isopropylacrylamide (NIPAM), N,N’-methylene-bis-acrylamide (bis-AMD), 2,2′-diethoxyacetophenone (DEAP) were purchased from Sigma Aldrich. Tetramethylethylenediamine (TEMED) and Acrylamide Polymerization (APS) were purchased from Bio-Rad. PTFE was purchased from 3 M (300LSE). Pressure Sensitive Vinyl (PSV) film (9786) was purchased from 3 M also. Nano pure deionized (DI) water (18.1 MΩ-cm) was produced in-house.

#### Micro post array fabrication

3D micro post array (MPA) was designed by 3D AutoCAD. The designed structure was printed by a 3D printing machine (Ultimaker 3) with extreme high resolution mode.

#### PTFE structure fabrication

In order to fabricate the PNIPAM hydrogel structure, confinement channels with desired shapes and dimensions were made using PTFE frame. Pore arrays in each PTFE film were designed by AutoCAD and fabricated by laser cutter (Versa laser cutter). The PTFE sheets are assembled by attachment of PSV film (10 μm) between each layer of PTFE. To avoid wrinkle and bubble between bonding, additional pressing treatment was followed by each bonding step. The adhesive layer of PTFE is firmly attached onto the PTFE cover layer after curing of NIPAM in the pore.

#### NIAPM solution

NIPAM (5 g) and BIS (10 mg) are dissolved into DI H_2_O (15 mL) and sonicated for 90 min at 30 °C. TEMED (50 μL) is added to control degree of crosslinking and sonicated until it appears white and opaque. To initiate polymerization, the APS (500 μL) is added.

#### PNIPAM synthesis in the multi-dimensional PTFE structure

Micro post array (MPA) is prepared by using 3D printer (Ultimaker 2). The surface of MPA is attached with PDMS layer to ensure electrostatic bonding between MPA and next PTFE sheet. The PTFE sheets are assembled from layer 5 to layer 2 by using PSV film. Top surface of layer 2 is attached by PVA film and alternative surface is unaltered with the protection layer. NIPAM solution (NIPAM (400 mg) in 4 mL H_2_O is prepared and 4 mg of BIS is added into the NIPAM solution. To ensure initiation of polymerization, APS (400 μL) is added) is loaded into the pore structure. After thermal curing of NIPAM without drying, the protection layer of PVA on layer 2 is removed. Layer 1 is attached on the assembled PTFE structure. We deployed the assembled self-activated pore structure from MPA carefully. To avoid wrinkle and bubble between bonding, doctor blade treatment was followed by each bonding step. The adhesive layer of PTFE is firmly attached onto the PTFE cover layer after curing of NIPAM in the pore. The deployed self-activated pore structure from MPA is hydrated under conditions of 100% humidity to absorb enough water due the amine group of the PNIPAM. Finally, the pore in the self-activated pore structure closes automatically (Fig. 3).

### Numerical simulation

#### Heat transfer coupled mechanics model

We coupled the heat transfer model with the mechanics of the hydrogel.


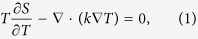


where *T* is the strain temperature, *S* is the strain of the hydrogel, *k* is the thermal conductivity. The mechanical strain of the hydrogel is dependent on temperature and the elastic property.


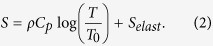


And *S*_*elast*_ can be defined as follows^35^,^36^,^41^:


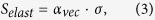


where σ is the stress vector. Each directional component can be composed, and *α*_*vec*_ is the thermal expansion coefficient vector. In case of an isotropic material, we can define the Selast as follows:


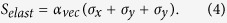


For the mechanical deformation, the equation (1) can be simplified as follows:


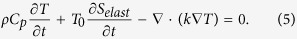


Using the above temperature-coupled mechanical model, temperature dependent volumetric strain and hydration are considered in the simulation. The hydration phenomenon is defined as a function of temperature based on published reports^35^,^37^,^42^,^43^.

#### Simulation method

The Finite element method (FEM, COMSOL, Multiphysics software) was used to model the self-actuation of the self-activated pore structure in water. This was done to achieve a solution to stress and strain dependent on temperature change. The mesh structure of the self-activated pore was constructed using hybrid mesh generation. The adaptive mesh was refined until the maximum electric field converged. We used the FEM primarily because of its ability to produce an adaptive mesh as required by the complex geometry of the pore with temperature change. It is also more advantageous than the finite difference time domain (FDTD) method which is good for rectangular geometry.

## Additional Information

**How to cite this article**: Park, Y. *et al*. Reversible Self-Actuated Thermo-Responsive Pore Membrane. *Sci. Rep.* **6**, 39402; doi: 10.1038/srep39402 (2016).

**Publisher's note:** Springer Nature remains neutral with regard to jurisdictional claims in published maps and institutional affiliations.

## Supplementary Material

Supplementary Information

Supplementary Video
