# Supplementary material for: Reversible Self-Actuated Thermo-Responsive Pore Membrane
Source: Sci Rep. 2016 Dec 19;6:39402. doi: 10.1038/srep39402 (PMC5171239; doi:10.1038/srep39402)
Supplement: Supplementary Information [file srep39402-s1.pdf]

## Supporting information

# Reversible Self-Actuated Thermo-Responsive Pore Membrane

**Younggeun Park[1,2], Maria Paz Gutierrez[5], and Luke P. Lee[1,2,3,4] ***

[1]Department of Bioengineering, University of California at Berkeley, California, 94720, USA.
[2]Berkeley Sensor and Actuator Center, University of California at Berkeley, California, 94720, USA.
[3]Department of Electrical Engineering and Computer Science, University of California at Berkeley, California, 94720, USA.
[4]Biophysics Graduate Program, University of California at Berkeley, California, 94720, USA.
[5]Department of Architecture, University of California at Berkeley, California, 94720, USA.

*Correspondence and requests for materials should be addressed to L.P.L (email: lplee@berkeley.edu)

**Contents**

Table S1. Geometric parameters and detailed dimension
Table S2. Values of physical and chemical properties for hydrogel simulation

Figure S1. Design parameters of the self-activated pore structure. (a) Geometric parameter definition of self-activated pore structure, (b) Computational mesh and (c) Flow chart for coupled simulation study between heat transfer and mechanics of the self-activated pore structure structure.
Figure S2. Pore structures; (a) SEM image of pore structure, (b) closed and opened pore images, and (c) cross sectional images of pore closed and opened.
Figure S3. Diameter and thickness effect on pore opening.
Figure S4. Pore membrane images at each bening angle.

Supporting video legend
Supporting Video: Reversible Self-Actuated Thermo-Responsive Pore

**Table S1**. Geometric parameters and detailed dimension

| *No* | $d_0$ | $d_1$ | $L_1$ | $L_M$ | $L_2$ | $d_r$ | *H* | *Scale* |
|---|---|---|---|---|---|---|---|---|
| **1** | 2000 | 2000 | 200 | 1000 | 200 | 1 | 0.2 | 1 |
| **2** | 3000 | 2000 | 200 | 1000 | 200 | 1.5 | 0.2 | 1 |
| **3** | 3500 | 2000 | 200 | 1000 | 200 | 1.75 | 0.2 | 1 |
| **4** | 5000 | 2000 | 200 | 1000 | 200 | 2.5 | 0.2 | 1 |
| **5** | 8000 | 2000 | 200 | 1000 | 200 | 4 | 0.2 | 1 |
| **6** | 3500 | 2000 | 200 | 1000 | 200 | 1.75 | 0.2 | 1 |
| **7** | 3500 | 2000 | 400 | 1000 | 400 | 1.75 | 0.4 | 1 |
| **8** | 3500 | 2000 | 1000 | 1000 | 1000 | 1.75 | 1 | 1 |
| **9** | 3500 | 2000 | 2000 | 1000 | 2000 | 1.75 | 2 | 1 |
| **10** | 3500 | 2000 | 4000 | 1000 | 4000 | 1.75 | 4 | 1 |
| **11** | 2000 | 1000 | 200 | 1000 | 200 | 2 | 0.2 | 0.5 |
| **12** | 3000 | 1500 | 200 | 1000 | 200 | 2 | 0.2 | 0.75 |
| **13** | 3500 | 2000 | 200 | 1000 | 200 | 1.75 | 0.2 | 1 |
| **14** | 5000 | 2500 | 200 | 1000 | 200 | 2 | 0.2 | 1.25 |
| **15** | 8000 | 4000 | 200 | 1000 | 200 | 2 | 0.2 | 2 |

**Table S2**. Values of physical and chemical properties for hydrogel simulation

| Property | Value | Unit |
| --- | --- | --- |
| Young's modulus, *E* | 157 | GPa |
| Density, *r* | 2330 | $Kg/m^3$ |
| Poisson's ratio, n | 0.3 | |
| Thermal expansion coefficient, *a* | $2.6 \times 10^{-6}$ | $K^{-1}$ |
| Specific heat, *Cp* | 700 | J/(Kg×K) |
| Thermal conductivity, *k* | 90 | W/(m×K) |
| Ambient and initial temperature, *T* | 300 | K |

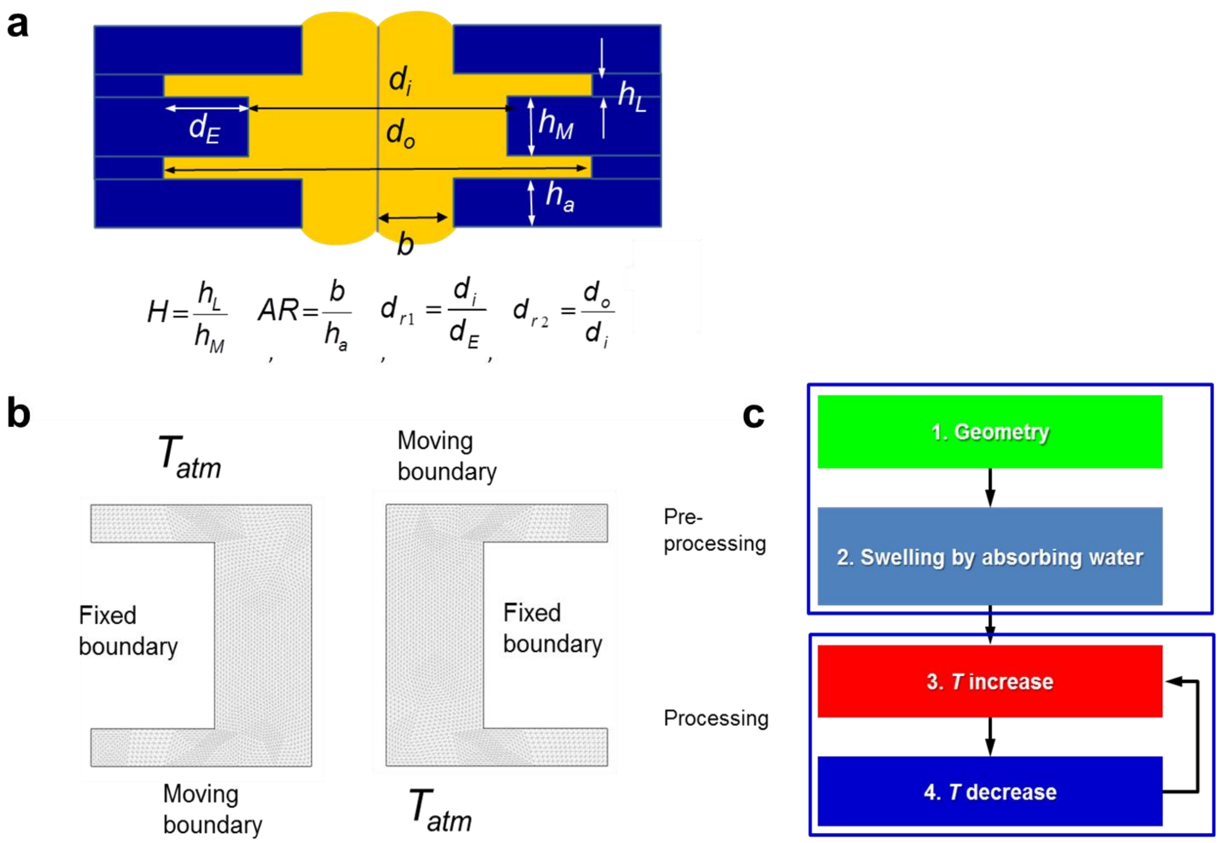


**Figure S1.** Design of self-activated pore structure and simulation method. (a) Definition of geometric parameters, (b) Computational mesh, and (c) Flow chart for coupled simulation study between heat transfer and mechanics of the self-activated pore structure structure.

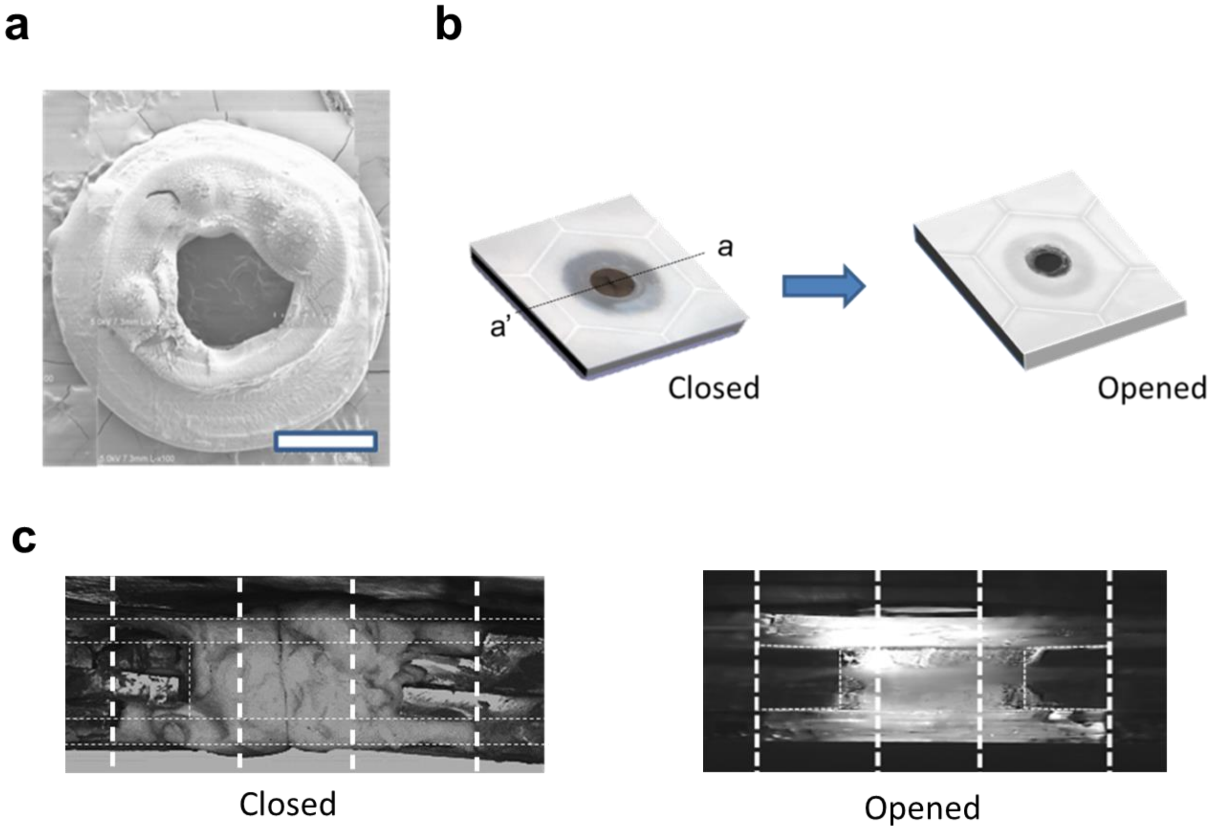


**Figure S2.** Pore structures; (a) SEM image of pore structure, (b) closed and opened pore images, and (c) cross sectional images of pore closed and opened.

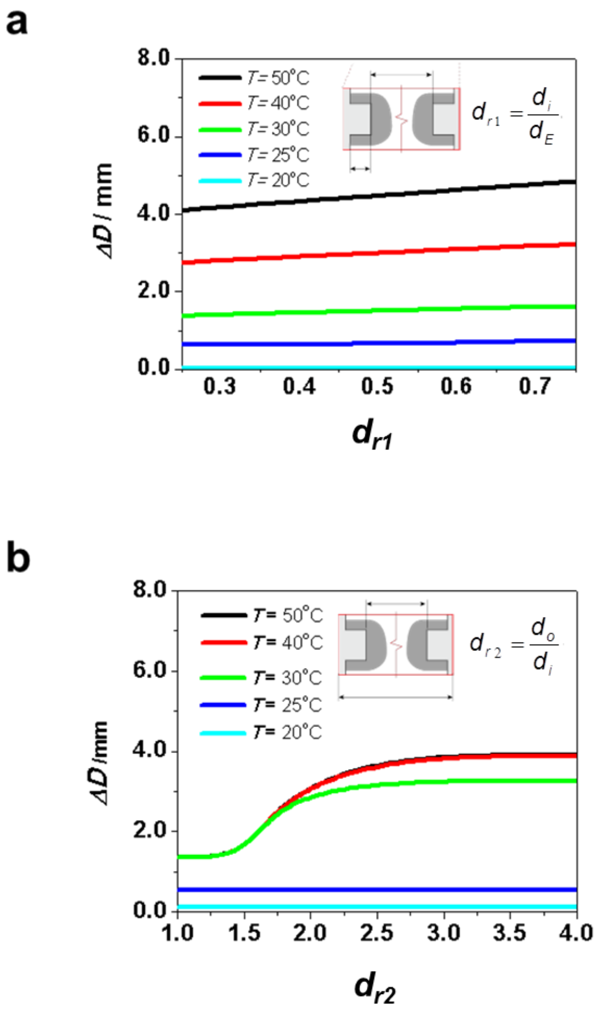


**Figure S3.** Diameter and thickness effect on pore opening. (a) Relative thickness effect on mechanical displacement change at $d_{r2}$ = 2.0, AR = 2.0 and $H$ = 1.0and (b) Relative diameter effect on mechanical displacement change at $d_{r1} = 0.75$, AR = 2.0 and $H$ = 1.0.

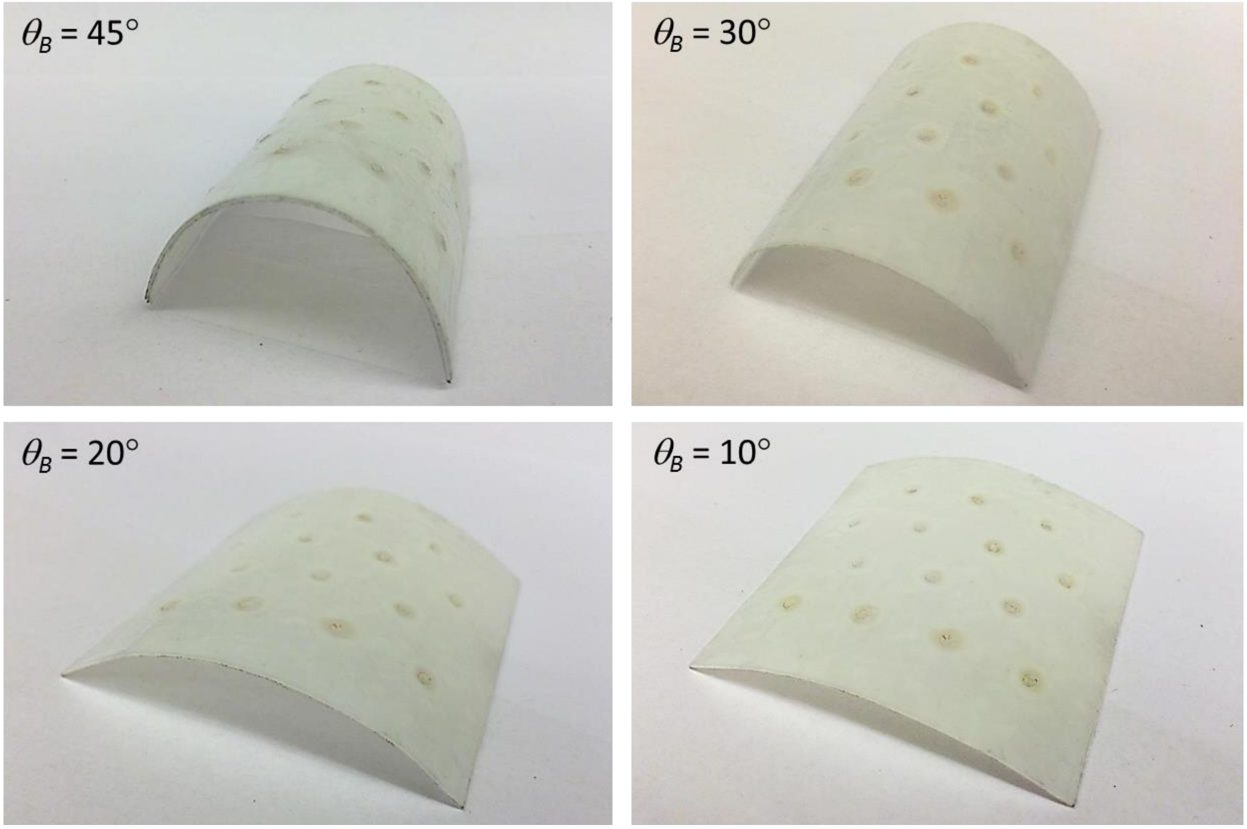


**Figure S4.** Pore membrane images at each bening angle.

**Supporting video legend**

Supporting Video: Reversible Self-Actuated Thermo-Responsive Pore

The reversible self-actuated rhermo-responsive pore is closed at T=20$^{o}$C and it is opened at T= 40$^{o}$C. The video clip shows that this pore open and close cycle is reversible.
